# Spillover effects of the COVID-19 pandemic on attitudes to influenza and childhood vaccines

**DOI:** 10.1186/s12889-023-15653-4

**Published:** 2023-04-25

**Authors:** Anna Soveri, Linda C. Karlsson, Jan Antfolk, Otto Mäki, Linnea Karlsson, Hasse Karlsson, Saara Nolvi, Max Karukivi, Mikael Lindfelt, Stephan Lewandowsky

**Affiliations:** 1 FinnBrain Birth Cohort Study, Institute of Clinical Medicine, University of Turku, Turun Yliopisto, 20014 Finland; 2 Department of Psychology, Åbo Akademi University, Turku, Finland; 3 Department of Psychology and Speech-Language Pathology, University of Turku, Turku, Finland; 4 Centre for Population Health Research, University of Turku and Turku University Hospital, Turku, Finland; 5 Department of Child Psychiatry, Turku University Hospital and University of Turku, Turku, Finland; 6 Department of Psychiatry, Turku University Hospital and University of Turku, Turku, Finland; 7 Department of Psychology and Speech-Language Pathology, Turku Institute for Advanced Studies, University of Turku, Turku, Finland; 8 Department of Adolescent Psychiatry, Turku University Hospital and University of Turku, Turku, Finland; 9 Department of Theological Ethics, Åbo Akademi University, Turku, Finland; 10 School of Psychological Science, University of Bristol, Bristol, UK; 11 School of Psychological Science, University of Western Australia, Perth, Australia

**Keywords:** Vaccine attitudes, COVID-19, Vaccinations, Vaccine hesitancy, Perceived risk, Perceived safety

## Abstract

The current study sought to determine whether public perceptions of other vaccines and diseases than COVID-19 have been impacted by the COVID-19 pandemic. We longitudinally examined whether there had been a change from before the COVID-19 pandemic to during the pandemic in: (a) influenza vaccination behaviour and intentions; (b) the perceived benefit of childhood vaccines and influenza vaccines; (c) the perceived safety of childhood vaccines and influenza vaccines; (d) the perceived severity of measles and influenza; and (e) trust in healthcare professionals in two samples of Finnish adults (*N* = 205 in Study 1 and *N* = 197 in Study 2). The findings showed that during the pandemic, more people than before had received or wanted to receive the influenza vaccine. The respondents also believed that influenza was more dangerous during the pandemic and that vaccinations were safer and more beneficial. On the other hand, for childhood vaccines only perceived safety increased. Finally, in one of the studies, people had more confidence in medical professionals during the pandemic than they had before. Together, these findings imply a spillover of the COVID-19 pandemic on how people view other vaccines and illnesses.

**Supplementary Information:**

The online version contains supplementary material available at 10.1186/s12889-023-15653-4.

The COVID-19 pandemic is a global health emergency that has changed our lives in many ways. At the heart of these changes lies the threat of a novel infectious disease. Over the course of the pandemic, we have experienced the detrimental consequences for individuals and society when the population is exposed to a virus against which there is no immunity. We have lived with restrictions implemented by governments to manage the spread of the disease [1]. We have experienced the overabundance of information on COVID-19 through news coverage and social media [2, 3]. Simultaneously, we have experienced the development and rollout of vaccines against COVID-19 [4] and witnessed the growing body of evidence indicating that the vaccines are safe and effective [5, 6]. Alongside these experiences, we have shaped our perceptions for example about the severity of COVID-19, the safety and benefit of the vaccines that protect against COVID-19, and the trustworthiness of the authorities in handling the crisis. Perceptions about disease risk, vaccine safety, and vaccine benefit, as well as trust in the actors involved in vaccinations, are factors that are known to be generally associated with vaccination decision-making [7–9]. Based on this, it has recently been suggested that the experience of the pandemic, and the perceptions shaped because of the pandemic, may also have altered people’s perceptions of other diseases and vaccines [10] – so-called spillover effects. From this perspective, the COVID-19 pandemic is a natural quasi-experiment that is altering several known psychological determinants of vaccine acceptance. To the best of our knowledge, the present study is the first to use data from the same participants before and during the COVID-19 pandemic to longitudinally investigate spillover effects of the pandemic on attitudes to influenza and childhood vaccines.

Previous evidence regarding spillover effects of health crises, such as outbreaks of infectious diseases, on people’s attitudes to other vaccines and diseases is scarce, and relates to the novel influenza A (H1N1) virus that began spreading around the world in the spring of 2009 [11]. One of the vaccines against H1N1 (Pandemrix) caused considerable controversy due to its association with an increased risk of narcolepsy [12]. A qualitative study consisting of interviews with parents conducted 2016–2019 in Finland showed that the adverse effects of the vaccine resulted in distrust in health authorities and vaccines in some individuals [13]. Spillover effects of the influenza A (H1N1) pandemic on other vaccines have been investigated also in an Australian study that compared parental attitudes towards influenza vaccines and childhood vaccines before and after the pandemic [14]. That study showed no change in attitudes to childhood vaccines, but there was an increase in negative attitudes to seasonal influenza vaccines after the pandemic, possibly due to febrile adverse effects attributed to a trivalent influenza vaccine used in 2010 against the virus.

Even though several studies have compared people’s vaccine attitudes or willingness to take vaccines before vs. during the COVID-19 pandemic [15–20], longitudinal evidence from the same people before and during the pandemic, is still lacking. Most previous studies suggest an increase in positive vaccine attitudes or willingness to get vaccinated from before the pandemic to during the pandemic (however, see [19, 20]). For example, retrospective reports in cross-sectional samples from six countries (US, Canada, Israel, Japan, Spain, Switzerland) during the first wave of the COVID-19 pandemic (March-June, 2020), suggested that parents were more willing to let their children receive the influenza vaccine during the pandemic compared to before it [16]. A study based on cross-sectional surveys with pregnant women in Italy before the pandemic and during the COVID-19 pandemic in November 2020-January 2021, showed that the trust in vaccine safety was higher during the pandemic than before [17]. Also, a cross-sectional study investigating vaccine attitudes in 28 European countries in 2018 and again during the first wave of the pandemic in March 2020 [15] indicated that people’s confidence in the safety and perceived importance of the measles, mumps, and rubella (MMR) vaccine and the influenza vaccine were higher during the pandemic in most of the included countries. The differences between the two time points were larger for the perceived safety and importance of influenza vaccines than for the perceived safety and importance of the MMR-vaccine. A more recent report from the same project that included an additional data collection conducted between March and August 2022, however, showed that people’s confidence in the MMR vaccine and influenza vaccine was higher in 2022 than in 2018, but had declined from 2020 [18]. A similar change can be seen in a study conducted in the US using cross-sectional survey data collected from parents [10]. The results from this study indicated that there was a decline in negative vaccine attitudes from before the pandemic to early pandemic in April-July 2020, but that this change disappeared later the same year. When it comes to trust in the actors involved in vaccinations, a longitudinal study with cross-sectional samples from 113 countries showed that people’s trust in doctors and nurses had increased on a global level from 2018 to late 2020 [21]. There have also been considerable increases in people’s trust in science and scientists in many countries [21–23].

In the present study, we attempted to shed more light on the possible spillover from the pandemic on people’s attitudes to childhood and influenza vaccines, risk perceptions of measles and influenza, and trust in health professionals, by using a longitudinal design with data from the same individuals before and during the pandemic. The previous studies investigating vaccine attitudes are either cross-sectional studies relying on retrospective reports collected at a single time point or cross-sectional studies with different samples before and during the pandemic. The longitudinal approach employed in the present study reduces the risk of recall bias and rules out explanations related to having unbalanced groups at the two testing points.

The first aim of the current study was to investigate if people’s influenza vaccination intentions and behavior changed from before to during the pandemic. Based on the previous study [16], we expected that more people have been vaccinated or have wanted to get vaccinated against influenza during the pandemic. In addition to influenza vaccination behavior and intentions, we explored possible changes in: (1) the perceived benefit of childhood vaccines and influenza vaccines, (2) the perceived safety of childhood vaccines and influenza vaccines, (3) the perceived severity of measles and influenza, and (4) trust in health care professionals and health authorities in vaccine-related matters. Drawing on the previous research [15, 17, 21], we expected that people perceive the benefit and safety of vaccines to be higher during the pandemic than before the pandemic, especially concerning influenza vaccines. Furthermore, as COVID-19 has frequently been compared to influenza [24, 25], we also expected spillover effects to be larger for influenza vaccines than for childhood vaccines. We finally hypothesized that trust in health care professionals will be higher during the pandemic than before. As there was no previous research investigating whether high threat of one disease increases the perceived threat of other diseases, our approach concerning the potential changes in the perceived severity of measles and influenza was exploratory.

Two samples of Finnish adults were included in the current study. In the following, the studies will be reported based on completion order and not on commencement. In both samples, the first data collection was conducted before the COVID-19 pandemic began. In Study 1, the first data collection took place in 2019 and the second one during the first wave of the pandemic in April 2020, before any COVID-19 vaccines were available. In Study 2, the first data collection was conducted in 2018 and the second one three years later, in June and July 2021 (see Fig. 1). That the data collections took place at different time points during the pandemic may be relevant, as a research shows that people’s willingness to take the influenza vaccine decreased during 2020 in the US and general vaccine attitudes became more negative [26]. Furthermore, trust in authorities in the US increased when COVID-19 vaccinations had started [27]. Based on this, it is possible that the change in vaccine attitudes, vaccination behavior and trust will be larger in Study 2, where the data collection took place after the COVID-19 vaccinations had commenced.

## Study 1

### Method

#### Participants and procedure

**Fig. 1:**
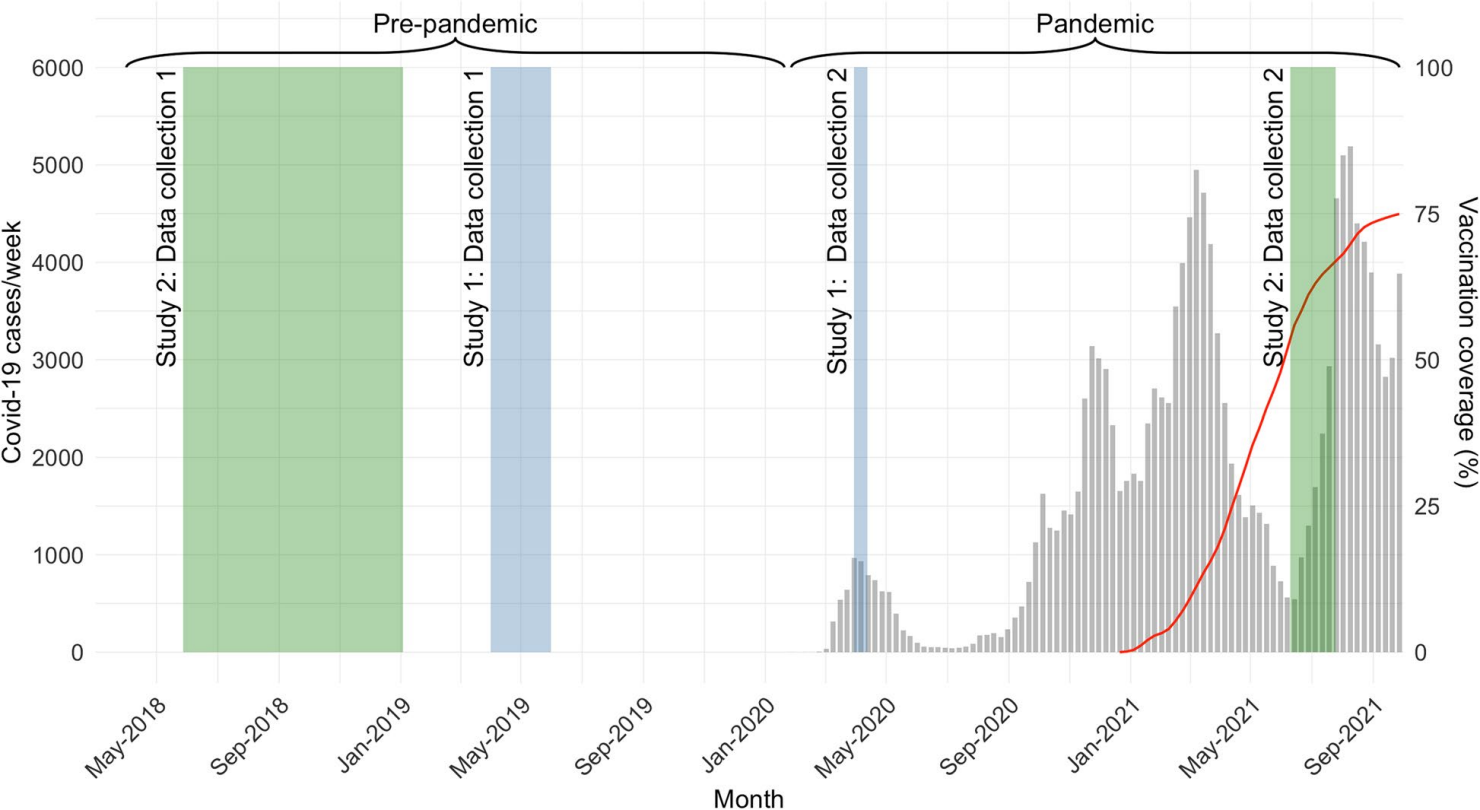
Timeline of Data Collections, COVID-19 Cases and COVID-19 Vaccination Coverage in Finland. *Note. *Weekly number of new COVID-19 cases (grey bars) and vaccination coverage for the first dose of a COVID-19 vaccine (red line) in the total population in Finland together with the timeline for the two data collections in Study 1 (blue fields) and Study 2 (green fields)

In April 2019, 5000 18–65-year-old individuals living in the Pietarsaari region in Finland were sent a letter with an invitation to participate in an electronic survey on vaccine attitudes and behaviors. The individuals were randomly selected from a register that contains information on all individuals living in Finland (Finnish Population Information System [28]). The sampling was done using proportional stratified random sampling based on gender and language (Finnish and Swedish) within each municipality in the area. The Pietarsaari region was chosen because of a lower uptake of several vaccines included in the national vaccination program compared to other Finnish regions [29]. Altogether, we received 1139 (22.8%) responses and out of those, 335 (29.4%; 6.7% of the whole invited sample) provided an email address and gave us permission to contact them again. These 335 respondents were invited via email to take part in a survey on the COVID-19 pandemic between March 30th and April 12th, 2020 (see Fig. 1). The final sample included in the present study consisted of the 205 (61.2%; 4.1% of the sample targeted in 2019) individuals who had replied to both surveys (see Table 1 for sample descriptives). Compared to the population structure in the Pietarsaari area [30], women were overrepresented and 50-65-year-olds underrepresented in the present sample. Also the education level was higher in the present sample [31]. The respondents received no compensation for participating.

**Table 1:** Sample descriptives at time point 1 for Study 1 and Study 2

Variable	Study 1		Study 2	
	n	%	n	%
Gender				
Male	63	30.7	44	22.3
Female	141	68.8	153	77.7
Do not want to report	1	0.5	-	-
Age				
18–29	34	16.6	5	2.5
30–39	64	31.2	120	60.9
40–49	46	22.4	70	35.5
50–59	29	14.1	2	1.0
60+	31	15.1	0	0.0
Education				
Basic/Upper secondary	96	46.9	43	21.8
Tertiary	105	51.1	147	74.6
Other	4	2.0	7	3.6
Children				
Yes	145	70.7	197	100.0
No	60	29.3	0	0.0
Language				
Finnish	35	17.1	161	81.7
Swedish	170	82.9	36	18.3

#### Measures

All items used in Study 1 and Study 2 can be found in Table S1.

##### Intentions to take an influenza vaccine

At both time points, we measured intentions to take the influenza vaccine with the question: ”Will you take the influenza vaccine during the upcoming season?” The years for the upcoming season were specified in parenthesis (i.e., 2019–2020 and 2020–2021, respectively). The response alternatives were: ”no”, ”yes” and ”I do not know”.

##### Perceived benefits of vaccines

Five items measured the perceived benefits of childhood vaccines (e.g., “Vaccinating children with childhood vaccines protects others, because it stops the spread of the diseases” and “Childhood vaccines are not necessary because good hand hygiene will make the diseases disappear from society” [reversed statement]). Four statements measured the perceived benefit of influenza vaccines (e.g., “Getting vaccinated against influenza, protects others from catching the disease” and “A good hand hygiene and other preventive measures are enough to avoid the flu without vaccination” [reversed statement]). All statements were administered at both time points. The respondents answered all these items on a scale from 1 (completely disagree) to 6 (completely agree).

##### Perceived safety of vaccines

The perceived safety of vaccines was measured with four items each for childhood (e.g., “Childhood vaccines are safe” and “The benefits of childhood vaccines are greater than the risk of side effects”) and influenza vaccines (e.g., “Influenza vaccines are safe” and “The risk of side effects weighs more than the benefits of influenza vaccines” [reversed statement]). All statements were administered at both time points. The respondents answered all these items on a scale from 1 (completely disagree) to 6 (completely agree).

##### Perceived severity of disease

One statement was included in the surveys to measure the perceived severity of measles and influenza at both time points. For measles, it was: “Measles is a very serious disease”, and for influenza it was: “It is not worth getting the influenza vaccine, as the influenza symptoms are not serious” (reversed statement). The respondents answered both items on a scale from 1 (completely disagree) to 6 (completely agree).

##### Trust

Trust in health professionals was measured with five statements that were administered at both time points (e.g. “I trust the information I receive from doctors about vaccines” and “Health professionals would not recommend vaccines that are unsafe”). The respondents answered on a scale from 1 (completely disagree) to 6 (completely agree).

#### Statistical analyses

The McNemar-Bowker Test of Symmetry [32], which is suitable for analyzing the relationship between categorical variables in a repeated-measures design, was used to detect pre-pandemic vs. pandemic differences in the intentions to take influenza vaccines. For all other constructs where the aim was to use composite scores, we first used McDonald’s Omega to estimate internal consistency. For scales with internal consistencies of ≥ 0.6 before and during the pandemic, we then created composites by calculating an average of the items within each construct. After this, paired-samples *t*-tests were conducted to examine whether there was a difference between the two time points. To check for gender differences, we also ran separate McNemar-Bowker Tests of Symmetry and paired-samples *t*-tests for male and female respondents. One individual had not reported their gender and is therefore not included in the gender-specific analyses. IBM SPSS Statistics 27 was used for all analyses and computations.

### Results

Means and standard deviations for all items in Study 1 and Study 2 are reported in Tables S2, S3 and S4. The analyses conducted by gender are reported in Tables S5 and S6.

#### Internal consistency

McDonald’s Omega for the four items planned to measure perceived safety of influenza vaccines could not be estimated, because the item: “The risk of side effects outweighs the benefits of influenza vaccines” had covariances close to zero with the other items. After excluding that item, McDonald’s Omega was good both before and during the pandemic. We thus used a composite with three items in the analyses. Most other composites showed acceptable to excellent internal consistency. The only exception was the composite for the perceived benefit of childhood vaccines during the pandemic. See Table 2 for estimates of internal consistency.

**Table 2:** Internal consistency for the composites in Study 1 and Study 2

Study				Internal Consistency	
		*n*	Items *n*	Before	During
1	Childhood vaccines: Benefit	201	5	0.823	0.657
1	Childhood vaccines: Safety	195	4	0.830	0.789
1	Influenza vaccines: Benefit	201	4	0.849	0.818
1	Influenza vaccines: Safety^a^	193	3	0.852	0.833
1	Trust	199	5	0.955	0.959
2	Childhood vaccines: Benefit	181	5	0.607	0.623
2	Childhood vaccines: Safety^b^	177	4	0.630	0.582
2	Childhood vaccines: Safety^c^	177	3	0.634	0.735
2	Influenza vaccines: Benefit	189	2	0.523	0.378
2	Influenza vaccines: Safety	186	2	0.769	0.770
2	Trust	189	3	0.848	0.852

The Spearman-Brown coefficient was used for scales with two items. McDonald’s omega was employed for all other scales

^a^Reliability after excluding item: “The risk of side -effects outweighs the benefits of influenza vaccines”

^b^Reliability with all four items

^c^Reliability after excluding item: “The risk of side-effects outweighs the benefits of childhood vaccines”

#### Intentions to take an influenza vaccine

The results of a McNemar-Bowker Test of symmetry showed that the intentions to take an influenza vaccine had not changed from before to during the pandemic, *X*^*2*^(3, *N* = 201) = 2.80, *p* = .423. This was the case also in the separate analyses for men (3, *N* = 62) = 0.76, *p* = .860) and women (3, *N* = 138) = 3.33, *p* = .344).

#### Perceived benefit of vaccines

The paired samples *t*-test showed that there was a change in the perceived benefit of childhood vaccines from before to during the pandemic, *t*(200) = 2.02, *p* = .045, *d* = 0.14, 95% CI[0.00, 0.28], such that people perceived childhood vaccines as *less* beneficial during the pandemic than before. The by-gender analyses indicated that this change was seen only in men. The analysis on the perceived benefit of influenza vaccines revealed a change in the opposite direction, *t*(200) = -6.56, *p* < .001, *d* = − 0.46, 95% CI[-0.61, − 0.32], such that the respondents considered the benefits of influenza vaccines to be greater during the pandemic than before. This was the case for both men and women.

#### Perceived safety of vaccines

There was no statistically significant change in the participants’ perception of the safety of childhood vaccines, *t*(194) = -1.61, *p* = .109, *d* = − 0.12, 95% CI [-0.26, 0.03]. Among only men, however, the results showed an increase in perceived safety from before the pandemic to during the pandemic.

The results further showed an increase in the perceived safety of influenza vaccines from before to during the pandemic, *t*(192) = -2.08, *p* = .039, *d* = − 0.15, 95% CI [-0.29, − 0.01]. The by-gender analyses showed that this change was statistically significant only in men.

#### Perceived severity of disease

The paired-samples *t*-tests showed that there were no changes in the perceived severity of measles from before to during the pandemic, *t*(201) = 0.47, *p* = .643, *d* = 0.03, 95% CI[-0.11, 0.17]. This was the case also for the gender-specific analyses. For influenza vaccines, on the other hand, there was a before vs. during pandemic difference, *t*(204) = -3.14, *p* = .002, *d* = − 0.22, 95% CI [-0.36, − 0.08], indicating that people considered it more important to get vaccinated due to the severity of influenza during the pandemic than before. This change was seen in both men and women.

#### Trust

The results showed a change in trust from before to during the pandemic, *t*(198) = -4.33, *p* < .001, *d* = − 0.31, 95% CI [-0.45, − 0.17], stemming from the fact that trust in health care professionals and authorities, when it comes to vaccine-related matters, was higher during the pandemic than before. This change was seen both in men and women.

### Discussion

The results of Study 1, in which the second data collection took place before COVID-19 vaccines had become available, showed that the respondents perceived influenza to be more severe, and the influenza vaccines to be more beneficial and safe, during the pandemic. Despite this, people’s intentions to take an influenza vaccine during the next influenza season had not increased. The perceived safety of childhood vaccines had not changed, but contrary to our hypothesis, people perceived the benefit of childhood vaccines to be slightly smaller during the pandemic than before. It is important to note that the internal consistency of the items measuring the perceived benefit of childhood vaccines was low at the second time point, possibly hampering the results. Furthermore, this result was seen only in men. The perceived severity of measles had not changed from before to during the pandemic. Finally, participants had higher trust in health care professionals during the pandemic than before. The results from the gender-specific analyses indicated that even if the perceived safety of childhood vaccines had not changed in the whole sample, men perceived the safety of childhood vaccines to be higher during the pandemic than before. Furthermore, the increase in perceived safety of influenza vaccines was seen only in men.

In Study 2, we investigated whether the pattern of changes from before the pandemic to during the pandemic would be the same as in Study 1, in a different sample of Finnish adults. In Study 2, the second data collection took place in 2021, half a year after COVID-19 vaccinations had commenced and more than 50% of the Finnish population had received the first dose (see Fig. 1). We expected that the change in attitudes to influenza and childhood vaccines would be larger in Study 2 than in Study 1, as previous research has shown that people’s hesitancy towards COVID-19 vaccines started to decline after rollout [27].

## Study 2

### Method

#### Participants and procedure

The participants in Study 2 were parents from the FinnBrain Birth Cohort Study [33] (hereafter called Finnbrain), which is an ongoing longitudinal project investigating child development. The FinnBrain population consists of parents recruited at three maternal welfare clinics in southwestern parts of Finland (the Turku region and the Åland islands). Apart from younger mothers being slightly underrepresented, the cohort is representative for the source population of families expecting children [33].

The first of the two data collections included in the present study took place before the pandemic, between May and December 2018 (see Fig. 1), when 3401 FinnBrain parents with at least one child younger than 4.5 years^1^, were sent an invitation letter with a participant-specific web-address to an online survey. Altogether, 833 parents responded. After excluding 50 parents because of missing informed consent, and 13 because they did not give permission to connect their responses to data previously collected about them, the sample consisted of 770 parents. The second data collection took place after vaccine rollout, between June and July, 2021. A letter with a link to an online survey was sent out to all FinnBrain parents who had answered a previous survey in May 2020^2^ about the COVID-19 pandemic. Five of those individuals were excluded, because they had withdrawn from the study. Altogether, 851 parents were invited. We received 419 responses and of those, our final sample consisted of 197 parents who had responded also to the first data collection in 2018 (see Table 1 for sample characteristics). In this final sample, women were overrepresented (78%) compared to the gender distribution among the parents in FinnBrain (66% mothers; [33]). The respondents again received no compensation for participating.

### Measures

#### Past influenza vaccination behavior

At both time points, we measured past influenza vaccinations with the question: ”Did you take the influenza vaccine during the previous season?”. The years for the previous season were specified in parenthesis (“2017–2018” and “2020–2021”, respectively). There was a difference in response alternatives between the two time points. At the first time point, the alternatives were ”no” and ”yes”. Because there were not enough influenza vaccines during the 2020–2021 season, the response alternatives were ”no”, ”yes” and ”I wanted to get vaccinated, but there were no vaccines available”. The two latter response alternatives were coded as ”yes”.

#### Perceived benefit of vaccines

At both time points, five items were used to measure the perceived benefit of childhood vaccines. The statements were almost the same as in Study 1. In addition to smaller discrepancies concerning polarity or word order, there were some differences in the specific diseases (“measles” or “childhood diseases”) that were mentioned in the statements (See Table S1 for the surveys). The biggest difference was that in Study 2, one statement was formulated to concern vaccines in general instead of childhood vaccines (“It is better to get immunity through the childhood diseases the vaccines are meant for, than through vaccines” in Study 1 and “It is better to get immunity through the disease than through the vaccine” in Study 2). In Study 2, two items were available to measure the benefit of influenza vaccines at both time points in this sample: “A good hand hygiene and other preventive measures are enough to protect against influenza without vaccination” and “Influenza vaccines offer effective protection against the disease”. These statements were also included in Study 1. The participants were asked to indicate their agreement with each statement on a scale from 1 (completely disagree) to 4 (completely agree).

#### Perceived safety of vaccines

The four statements that were used in Study 2 to measure the perceived safety of childhood vaccines at both time points were almost the same as in Study 1. The biggest difference was that two items were formulated to concern vaccines in general in Study 2 and childhood vaccines in Study 1. The perceived safety of influenza vaccines was measured with the following two items that were also included in Study 1: “Influenza vaccines are safe” and “The risk of side effects outweighs the benefits of influenza vaccines”. The respondents answered all statements on a scale from 1 (completely disagree) to 4 (completely agree).

#### Perceived severity of disease

The same statements as in Study 1 were used to measure the perceived severity of measles and influenza at both time points. The respondents answered on a scale from 1 (completely disagree) to 4 (completely agree).

#### Trust

Trust in health professionals was measured at both time points with three statements that were almost the same as in Study 1. The discrepancies concerned the type of health care professionals specified in the statements (“medical doctors’’ or “health care workers”). The respondents answered on a scale from 1 (completely disagree) to 4 (completely agree).

#### Statistical analyses

We used McDonald’s Omega to estimate the internal consistency of constructs with ≥ 3 items. The Spearman-Brown coefficient was used to test the internal consistency of the two items measuring perceived benefit of influenza vaccines and the two items measuring perceived safety of influenza vaccines. We again used paired-samples *t*-tests and the McNemar Test (instead of the McNemar-Bowker Test of Symmetry due to only two categories) to examine whether there was a difference between the two time points. By gender paired samples *t*-tests were again conducted. IBM SPSS Statistics 27 was used for all analyses.

### Results

#### Internal consistency

The Spearman-Brown coefficient indicated that the internal consistency of the two items measuring perceived benefit of influenza vaccines was poor both before and during the pandemic. We, therefore, chose to analyze the two items (“A good hand hygiene and other preventive measures are enough to protect against influenza without vaccination” and “Influenza vaccines offer effective protection against the disease”) separately. McDonald’s Omega suggested poor internal consistency of the items planned to measure the perceived safety of childhood vaccines at the second time point. Because the analyses indicated that deleting the item: “The risk of side-effects outweighs the benefits of childhood vaccines”, would result in acceptable internal consistency, we excluded this item from the composite. A possible reason for poor internal consistency of the items measuring perceived safety of childhood vaccines was that two of the statements concerned vaccines in general, instead of childhood vaccines in particular (as in Study 1). The internal consistencies of the other composites varied from questionable to good (see Table 2).

#### Past influenza vaccination behavior

The results of the McNemar Test showed that there was a difference in past influenza vaccination behavior before and during the pandemic, *X*^*2*^(*N* = 191) = 7.20, *p* = .007, such that many people who did not take the influenza vaccine during the season 2017–2018, took it, or wanted to take it but no vaccines were available, during the season 2020–2021. The by-gender analyses revealed that the change was seen in women, *X*^*2*^(*N* = 148) = 8.03, *p* = .005, but not in men, *X*^*2*^(*N* = 43), *p* = 1.000.

#### Perceived benefit of vaccines

The paired-samples *t*-test showed that there was no change from before to during the pandemic in how beneficial people perceived childhood vaccines, *t*(180) = 0.05, *p* = .959, *d* = 0.00, 95% CI [-0.14, 0.15]. This was the case also when men and women were analyzed separately. The perceived benefit of influenza vaccines compared to a good hand hygiene and other preventive measures, was, however, higher during the pandemic than before, *t*(189) = -2.62, *p* = .010, *d* = − 0.19, 95% CI [-0.33, − 0.05]. Also the perceived efficacy of the influenza vaccine in protecting against the disease was higher during the pandemic, *t*(189) = -4.61, *p* < .001, *d* = − 0.33, 95% CI [-0.48, − 0.19]. The by-gender analyses revealed that the change in these two variables happened only in women.

#### Perceived safety of vaccines

The paired-samples *t*-tests showed that people considered both childhood vaccines,* t*(176) = -5.85, *p* < .001, *d* = − 0.44, 95% CI[-0.59, − 0.29], and influenza vaccines, *t*(185) = -6.70, *p* < .001, *d* = − 0.49, 95% CI [-0.64, − 0.34], as safer during the pandemic than before. The change in both variables was statistically significant in both men and women.

#### Perceived severity of disease

There was no difference in how severe people perceived measles before and during the pandemic, *t*(188) = -0.97, *p* = .333, *d* = − 0.07, 95% CI[-0.21, 0.07]. No difference emerged in the gender-specific analyses either. The perceived importance of the influenza vaccine due to the severity of influenza, on the other hand, increased from before the pandemic to during the pandemic, *t*(188) = -2.61, *p* = .010, *d* = − 0.19, 95% CI [-0.33, − 0.05]. The by-gender analyses revealed a statistically significant change in men but not in women.

#### Trust

The paired-samples *t*-test showed that the degree of trust people felt towards health care professionals had not changed from before the pandemic to during the pandemic, *t*(188) = -1.06, *p* = .292, *d* = − 0.08, 95% CI [-0.22, 0.07]. This was the case also when the genders were analyzed separately.

### Discussion

The results of Study 2, where the second data collection was conducted approximately six months after the vaccinations had commenced, showed that people perceived influenza vaccines as more beneficial and safe during the pandemic than before the pandemic. They also perceived influenza as more severe during the pandemic. The results further showed that more people had taken the influenza vaccine (or had wanted to take the vaccine, but no vaccines were available) during the pandemic than before the pandemic, but as the response alternatives were different at the two time points, this result should be interpreted with caution. There was also an increase in the perceived safety of childhood vaccines from before the pandemic to during the pandemic. No change was seen in the perceived severity of measles, perceived benefit of childhood vaccines or trust in health care professionals. The results from the separate analyses for men and women revealed that the change in past influenza vaccination behavior and perceived benefit and efficacy of influenza vaccines was seen only in women, while the change in perceived severity of influenza was seen only in men. Important to note is, however, that the number of men in Study 2 was rather low (*n* = 44).

## General discussion

In two studies, we longitudinally tested the hypothesis that the COVID-19 pandemic has altered people’s (1) attitudes to other vaccines and diseases other than COVID-19, (2) influenza vaccination behavior and intentions, and (3) trust in health care professionals in vaccine-related matters. The samples in the two studies were Finnish adults. One of the samples was recruited based on stratified random sampling from a region with suboptimal vaccine uptake (Study 1) and the other one was recruited from a birth cohort study with parents living in southwestern parts of Finland (Study 2). Within each of the two studies, we had information from before and during the pandemic from the same individuals.

The results of the two studies consistently indicated that participants had more positive attitudes towards the safety and benefit of influenza vaccines during the COVID-19 pandemic than before the pandemic. They also were more likely to consider influenza to be a serious disease during the pandemic.

When it comes to childhood vaccines, however, the pattern was less clear. The participants’ perceptions of the severity of measles had not changed from before the pandemic to during the pandemic in either of the studies. However, the perceived safety of childhood vaccines increased in Study 2 but not in Study 1. Furthermore, the results of Study 1 showed that the participants considered the benefits of childhood vaccines to be smaller during the pandemic than before. This unexpected effect was, however, very small and no change was observed in Study 2. These differences in results between the two studies may be related to the fact that the questions measuring safety and benefit of childhood vaccines differed between Study 1 and Study 2, as two items were formulated to concern vaccines in general in Study 2 and childhood vaccines in Study 1. Another possible explanation for the inconsistency between the two studies when it comes to childhood vaccines, is that while all respondents in Study 2 were parents of young children (all had children below the age of 4.5 years during the 2018 data collection), only 70% of the respondents in Study 1 reported having children when they filled out the first survey. Furthermore, only 37% in Study 1 had children in the age of 0–6 years, which is when most childhood vaccines are given. Childhood vaccines thus had greater immediate importance in the Study 2 sample, which might explain why some changes relating to childhood vaccines were seen in Study 1 but not in Study 2, and vice versa.

Taken together, the present results are in line with the previous cross-sectional study [15] when it comes to the increase in the perceived benefit and safety of influenza vaccines and perceived safety of childhood vaccines from before the COVID-19 pandemic to during the pandemic. That study, however, indicated that people’s attitudes had become more positive also to the benefits of the MMR-vaccine. In the present study, we investigated childhood vaccines as a group of vaccines rather than asking only about the MMR-vaccine. Important to note is also that the great majority of our respondents held very positive attitudes to the benefit of childhood vaccines already before the pandemic. This was true especially in Study 2, where for example all but seven individuals had a composite score that indicated that they more than somewhat agreed with the statements that postulated the benefit of childhood vaccines.

A possible reason for the fact that there were more spillover effects to influenza vaccines than childhood vaccines, is that COVID-19 has often been compared to influenza [24, 25]. Also, the importance of taking the influenza vaccine to avoid influenza and COVID-19 co-infection and to protect the capacity of the health care system, has been highlighted [34]. This has been emphasized also by Finnish health authorities [35]. In Finland, the COVID-19 pandemic resulted in a shortage of influenza vaccines due to an unprecedented demand. The fact that these recommendations came after the second data collection in Study 1 had already been completed (see Fig. 1), may explain why the present study showed no change in Study 1 in the participants’ intentions to take the influenza vaccine in the future. In Study 2, on the other hand, where the second data collection was performed in 2021, there was a change in past influenza vaccinations, stemming from the fact that a higher number of participants reported having taken, or having wanted to take, the previous influenza vaccine during the pandemic than before the pandemic. A previous cross-sectional study [16] conducted during the first wave of the pandemic in 2020, however, showed that people had been more willing to let their children receive the influenza vaccine during the pandemic than before the pandemic. Those results were based on retrospective reports collected at one time point.

Finally, the results of the present study are in line with the previous longitudinal study that showed that people’s trust in doctors and nurses had increased on a global level from 2018 to late 2020 [21], as trust in health care professionals had increased from before the pandemic to during the pandemic in Study 1. The fact that there was no change in trust in Study 2, where the data collection was conducted after vaccinations had commenced, is not in line with a previous study that indicated that trust in authorities had started to increase in the U.S. when COVID-19 vaccinations had begun [27]. A possible reason for the discrepancy between the previous study and the current one is that the previous study only focused on trust in the process that ensures that vaccines are safe. Our trust construct was broader, as we also measured to what degree the participants trusted the information given by health care professionals about vaccines. Important to note here is also that there were differences in the trust items between Study 1 and Study 2. In Study 1, we measured trust in several groups - nurses, doctors, health professionals and health authorities - while in Study 2, we only included health professionals and doctors. As people may trust some health professionals more than others, it is possible that the difference between the two studies in levels of trust before and during the pandemic is at least partly explained by differences in survey items.

There are limitations to the generalizability of the results in both samples. In Study 1, we used stratified random sampling when recruiting respondents for time point one. At time point two, the sample was recruited from those who responded to the first survey and who had given us permission to contact them again. This resulted in a low response rate compared to the original targeted population and a sample that deviated from the general population in the Pietarsaari area when it comes to gender, age and education. Furthermore, a previous study using approximately the same sample, indicated that although the respondents were recruited from an area with sub-optimal vaccine uptake, their willingness to be vaccinated was at the same level as in other Finnish samples [36]. This suggests a selection bias, where people with more positive attitudes to vaccines were more likely to take part in the study. In Study 2, the individuals were recruited from a birth cohort study, which limits the generalizability to the general population. Also, parents who are part of the birth cohort may have more positive attitudes to vaccines than the general population, as FinnBrain is a longitudinal study with several medical examinations and health-related surveys. Important to note also for Study 2, is that the response-rate was rather low, which might have resulted in sampling bias. Due to the overrepresentation of women both in Study 1 and Study 2, we ran all analyses separately for men and women. For most variables, the change from before the pandemic to during the pandemic was similar in men and women. But for example, in Study 1, men perceived vaccines safer during the pandemic than before, while no change was seen in the analyses with only women. Furthermore, in Study 2, there was an increase in the perceived benefit and efficacy of influenza vaccines only among women. The gender-specific analyses should, however, be interpreted with caution, due to the low number of men in both studies.

The COVID-19 pandemic is a complex event that has affected the lives of individuals in numerous ways. Therefore, it is difficult to draw conclusions on which aspects of the pandemic are behind the observed change in people’s vaccine attitudes and vaccination behavior. The purpose in the present study of categorizing the attitudes and perceptions into perceived benefits of vaccines, perceived safety of vaccines, perceived disease risk, and trust, was, nevertheless, to (indirectly) obtain information on whether the pandemic has changed some aspects of vaccination decision-making more than other aspects. The results, however, showed that all aspects related to influenza had changed from before to during the pandemic, in two different samples of Finnish adults, suggesting that the pandemic may have affected several factors that are generally important determinants of vaccine acceptance. The change was particularly large for the perceived benefit of influenza vaccines in Study 1 and perceived safety of childhood and influenza vaccines in Study 2, with effect sizes of almost moderate size. For example, the size of the change in the perceived benefit of influenza vaccines in Study 1 (*d* = − 0.46) suggests that there is a 68.7% chance that an individual’s score is higher during the pandemic than before the pandemic (we calculated Probability of Superiority for within-subjects design using the package RProbSup for R [37]). The practical relevance of a change of this size is high, due to the large threat of vaccine hesitancy to public health.

Taken together, the results suggest that people’s attitudes to vaccines – especially influenza vaccines – were more positive during the pandemic than before the pandemic. People also perceived influenza as a bigger threat than before the pandemic and have been more willing to get vaccinated against influenza. This study, thus, suggests that there has been a spillover of the pandemic on the public’s acceptance of other vaccines than COVID-19.

## Supplementary Information

**Additional file 1.**

**Additional file 2.**

**Additional file 3.**

**Additional file 4.**

**Additional file 5.**

**Additional file 6.**

## Data Availability

The option of making data openly available has not been included in the informed consent in Study 1, and therefore the data will be shared only upon request (anjoso@utu.fi). Due to Finnish federal legislation on personal data protection in medical research, the original research data in Study 2 cannot be made available online, but data can potentially be shared with Material Transfer Agreement. Requests and collaboration initiatives can be directed to the Board of the FinnBrain Birth Cohort Study.

## Footnotes

1 This population was chosen to avoid loading the parents with too many questionnaires at the same time. All parents with 5-year-old children received a extensive survey during that time.

2 In May 2020, all parents in the FinnBrain study (*N* = 5103) were invited to a survey with questions related to the COVID-19 pandemic.
