# Supplementary material for: Spillover effects of the COVID-19 pandemic on attitudes to influenza and childhood vaccines
Source: BMC Public Health. 2023 Apr 25;23:764. doi: 10.1186/s12889-023-15653-4 (PMC10126550; doi:10.1186/s12889-023-15653-4)
Supplement: Supplementary file 1 — Additional file 1. [file 12889_2023_15653_MOESM1_ESM.docx]

| **Table S1** | | |
| --- | --- | --- |
| *Statements Used to Measure the Outcome Variables in Study 1 and Study 2* | | |
| Study 1 | Study 2 | Abbreviation |
| Vaccination intentions | | |
| Will you take the influenza vaccine during the upcoming season? | - | IVSelf_NextSeason |
| Past vaccinations | | |
| - | Did you take the influenza vaccine during the previous season? | IVSelf_LastSeason |
| Vaccine benefit | | |
| Getting vaccinated against influenza, protects others from catching the disease. | - | Influ_Herd |
| Influenza vaccines offer effective protection against the disease | Influenza vaccines offer effective protection against the disease. | Influ_Effective |
| A good hand hygiene and other preventive measures are enough to avoid the flu without vaccination.* | A good hand hygiene and other preventive measures are enough to protect against influenza without vaccination.* | Influ_Hygiene^a^ |
| It is important to get vaccinated against influenza every year. | - | Influ_Important |
| Vaccinating children with childhood vaccines protects others, because it stops the spread of the diseases | Vaccinating healthy children helps to protect others by stopping the spread of disease. | Child_Herd |
| Childhood vaccines provide effective protection against diseases. | Childhood vaccines are effective in protecting against disease. | Child_Effective |
| Childhood vaccines are not necessary because good hand hygiene will make the diseases disappear from society.* | A good hygiene will make measles disappear from society – the vaccine is not necessary.* | Child_Hygiene^a^ |
| Children need to be vaccinated against measles even though the disease is no longer common in Finland. | Children need vaccines for diseases that are not common anymore. | Child_Uncommon |
| It is better to get immunity through the childhood diseases the vaccines are meant for, than through vaccines.* | It is better to get immunity through the disease than through the vaccine.* | Child_Immunity^a^ |
| Vaccine safety | | |
| Influenza vaccines cannot cause autism. | - | Influ_Autism |
| Influenza vaccines do not contain dangerous quantities of mercury. | - | Influ_Mercury |
| Influenza vaccines are safe. | The influenza vaccines are safe. | Influ_Safe |
| The risk of side effects outweighs the benefits of influenza vaccines.* | The risk of side effects outweighs the benefits of influenza vaccines.* | Influ_SidEff |
| Childhood vaccines cannot cause autism. | Vaccines can cause autism.* | Child_Autism^b^ |
| Childhood vaccines do not contain dangerous quantities of mercury. | Vaccines contain dangerous quantities of mercury.* | Child_Mercury^b^ |
| Childhood vaccines are safe. | Childhood vaccines are safe. | Child_Safe |
| The benefits of childhood vaccines are greater than the risk of side effects. | The risk of side effects outweighs the protective benefits of the childhood vaccines.* | Child_SidEff^b^ |
| Disease severity | | |
| Measles is a very serious disease. | Measles is a very serious disease. | Child_Serious |
| It is not worth getting the influenza vaccine, as the influenza symptoms are not serious.* | It is not worth getting the influenza vaccine, as the influenza symptoms are not serious.* | Influ_Serious^a^ |
| Trust | | |
| I trust the information on vaccines I get from nurses/health care providers. | I trust the information on vaccines I get from health professionals. | Trust_Professional |
| I trust the information I receive from doctors about vaccines. | - | Trust_Doctor |
| Health professionals would not recommend vaccines that are unsafe. | Doctors would not recommend vaccines that are unsafe. | Trust_Recommend |
| Health professionals take my questions about vaccines seriously. | Health professionals take my questions about vaccines seriously. | Trust_Questions |
| I trust the vaccine recommendations given by health authorities. | - | Trust_Authority |
| *Note.* Study 1 response scale: 1 (completely disagree) – 6 (completely agree), Study 2 response scale: 1 (completely disagree) – 4 (completely agree).  * Reverse coded item.  ^a^ Reverse coded item in Study 1 and Study 2.  ^b^ Reverse coded item in Study 2. | | |
