# Supplementary material for: Spillover effects of the COVID-19 pandemic on attitudes to influenza and childhood vaccines
Source: BMC Public Health. 2023 Apr 25;23:764. doi: 10.1186/s12889-023-15653-4 (PMC10126550; doi:10.1186/s12889-023-15653-4)
Supplement: Supplementary file 2 — Additional file 2. [file 12889_2023_15653_MOESM2_ESM.docx]

**Table S2**

*Intentions to Take the Influenza Vaccine in Study 1*

| Item | Pre-pandemic | | | Post-pandemic | | |
| --- | --- | --- | --- | --- | --- | --- |
|  | no (%) | yes (%) | do not know (%) | no (%) | yes (%) | do not know (%) |
| IVSelf_NextSeason | 82 (40.80) | 50 (24.88) | 69 (34.33) | 80 (39.80) | 58 (28.86) | 63 (31.34) |
