# Supplementary material for: Spillover effects of the COVID-19 pandemic on attitudes to influenza and childhood vaccines
Source: BMC Public Health. 2023 Apr 25;23:764. doi: 10.1186/s12889-023-15653-4 (PMC10126550; doi:10.1186/s12889-023-15653-4)
Supplement: Supplementary file 3 — Additional file 3. [file 12889_2023_15653_MOESM3_ESM.docx]

**Table S3**

*Past Influenza Vaccinations in Study 2*

| Item | Pre-pandemic | | Mid-pandemic | | |
| --- | --- | --- | --- | --- | --- |
|  | no (%) | yes (%) | no (%) | yes (%) | wanted to (%) |
| IV_Self_LastSeason | 82 (42.93) | 109 (57.07) | 63 (32.98) | 115 (60.21) | 13 (6.81) |
