# Supplementary material for: Spillover effects of the COVID-19 pandemic on attitudes to influenza and childhood vaccines
Source: BMC Public Health. 2023 Apr 25;23:764. doi: 10.1186/s12889-023-15653-4 (PMC10126550; doi:10.1186/s12889-023-15653-4)
Supplement: Supplementary file 4 — Additional file 4. [file 12889_2023_15653_MOESM4_ESM.docx]

| **Table S4** | | | | | | | | |
| --- | --- | --- | --- | --- | --- | --- | --- | --- |
| *Means and Standard Deviations for All Items in Study 1 and Study 2* | | | | | | | | |
| Item | Study 1 | | | | Study 2 | | | |
|  | Pre-pandemic | | Mid-pandemic | | Pre-pandemic | | Mid-pandemic | |
|  | Mean | SD | Mean | SD | Mean | SD | Mean | SD |
| Vaccine benefit | | | | | | | | |
| Influ_Benefit_Composite | 3.72 | 1.30 | 4.14 | 1.14 | - | - | - | - |
| Influ_Herd | 4.41 | 1.61 | 4.77 | 1.38 | - | - | - | - |
| Influ_Effective | 3.71 | 1.49 | 4.24 | 1.29 | 2.75 | 0.76 | 3.04 | 0.77 |
| Influ_Hygiene^a^ | 3.85 | 1.57 | 4.00 | 1.43 | 2.79 | 0.87 | 2.96 | 0.93 |
| Influ_Important | 2.89 | 1.63 | 3.53 | 1.58 | - | - | - | - |
| Child_Benefit_Composite | 5.67 | 0.62 | 5.60 | 0.56 | 3.73 | 0.33 | 3.73 | 0.35 |
| Child_Herd | 5.76 | 0.71 | 5.76 | 0.56 | 3.94 | 0.29 | 3.86 | 0.46 |
| Child_Effective | 5.72 | 0.69 | 5.71 | 0.61 | 3.75 | 0.52 | 3.82 | 0.48 |
| Child_Hygiene^a^ | 5.73 | 0.77 | 5.68 | 0.82 | 3.78 | 0.57 | 3.82 | 0.52 |
| Child_Uncommon | 5.70 | 0.80 | 5.65 | 0.88 | 3.69 | 0.52 | 3.66 | 0.56 |
| Child_Immunity^a^ | 5.46 | 1.02 | 5.20 | 1.29 | 3.48 | 0.66 | 3.49 | 0.70 |
| Vaccine safety | | | | | | | | |
| Influ_Safety_Composite | 4.96 | 1.10 | 5.09 | 1.02 | 3.11 | 0.78 | 3.44 | 0.69 |
| Influ_Autism | 4.95 | 1.34 | 5.01 | 1.27 | - | - | - | - |
| Influ_Mercury | 4.94 | 1.25 | 5.09 | 1.18 | - | - | - | - |
| Influ_Safe | 4.98 | 1.22 | 5.17 | 1.11 | 3.16 | 0.78 | 3.49 | 0.66 |
| Influ_SidEff^a^ | 3.79 | 1.82 | 3.61 | 1.91 | 3.06 | 0.96 | 3.38 | 0.88 |
| Child_Safety_Composite | 5.05 | 1.01 | 5.15 | 0.89 | 3.56 | 0.51 | 3.72 | 0.43 |
| Child_Autism^b^ | 4.67 | 1.53 | 4.67 | 1.45 | 3.47 | 0.74 | 3.72 | 0.56 |
| Child_Mercury^b^ | 4.82 | 1.40 | 5.03 | 1.32 | 3.63 | 0.67 | 3.76 | 0.55 |
| Child_Safe | 5.19 | 1.07 | 5.33 | 0.91 | 3.57 | 0.62 | 3.67 | 0.49 |
| Child_SidEff^b^ | 5.54 | 0.94 | 5.58 | 0.74 | 3.56 | 0.77 | 3.38 | 0.96 |
| Disease severity | | | | | | | | |
| Child_Serious | 5.41 | 0.94 | 5.38 | 0.83 | 3.59 | 0.68 | 3.65 | 0.62 |
| Influ_Serious^a^ | 3.98 | 1.49 | 4.28 | 1.32 | 3.20 | 0.81 | 3.37 | 0.82 |
| Trust | | | | | | | | |
| Trust_Composite | 5.11 | 1.16 | 5.33 | 0.97 | 3.47 | 0.61 | 3.50 | 0.62 |
| Trust_Professional | 5.09 | 1.30 | 5.29 | 1.02 | 3.50 | 0.70 | 3.55 | 0.66 |
| Trust_Doctor | 5.20 | 1.21 | 5.37 | 1.00 | - | - | - | - |
| Trust_Recommend | 4.99 | 1.34 | 5.24 | 1.15 | 3.34 | 0.73 | 3.40 | 0.75 |
| Trust_Questions | 5.07 | 1.23 | 5.31 | 1.05 | 3.56 | 0.69 | 3.56 | 0.72 |
| Trust_Authority | 5.22 | 1.20 | 5.45 | 1.00 | - | - | - | - |
| *Note.* Study 1 response scale: 1–6, Study 2 response scale: 1–4.  ^a^ Reverse coded item in Study 1 and Study 2.  ^b^ Reverse coded item in Study 2. | | | | | | | | |
