# Supplementary material for: Spillover effects of the COVID-19 pandemic on attitudes to influenza and childhood vaccines
Source: BMC Public Health. 2023 Apr 25;23:764. doi: 10.1186/s12889-023-15653-4 (PMC10126550; doi:10.1186/s12889-023-15653-4)
Supplement: Supplementary file 5 — Additional file 5. [file 12889_2023_15653_MOESM5_ESM.docx]

| **Table S5** |  |  |  |  |  |  |  |  |
| --- | --- | --- | --- | --- | --- | --- | --- | --- |
| *Means, Standard Deviations and Results of Paired-samples t-tests for Men and Women in Study 1* | | | | | | | | |
| Item |  |  |  |  |  |  |  |  |
|  | Men | | | | | | | |
|  |  | Pre-pandemic | | Mid-pandemic | |  |  |  |
|  | *n* | Mean | *SD* | Mean | *SD* | *t* | *p* | *d* |
| Child_Benefit_Composite | 63 | 5.65 | 0.43 | 5.52 | 0.61 | 2.25 | .028 | .28 |
| Influ_Benefit_Composite | 59 | 3.88 | 1.19 | 4.29 | 1.00 | -3.42 | .001 | -.45 |
| Child_Safety_Composite | 61 | 5.05 | 0.85 | 5.31 | 0.71 | -2.63 | .011 | -.34 |
| Influ_Safety_Composite^a^ | 63 | 4.98 | 1.01 | 5.23 | 0.88 | -2.75 | .008 | -.35 |
| Child_Serious | 62 | 5.32 | 0.90 | 5.31 | 0.76 | 0.17 | .867 | .02 |
| Influ_Serious^b^ | 63 | 3.83 | 1.50 | 4.22 | 1.30 | -2.01 | .049 | -.25 |
| Trust_Composite | 62 | 5.23 | 1.02 | 5.42 | 0.82 | -2.22 | .030 | -.03 |
|  | Women | | | | | | | |
|  |  | Pre-pandemic | | Mid-pandemic | |  |  |  |
|  | *n* | Mean | *SD* | Mean | *SD* | *t* | *p* | *d* |
| Child_Benefit_Composite | 137 | 5.68 | 0.69 | 5.64 | 0.53 | 0.89 | .374 | .08 |
| Influ_Benefit_Composite | 141 | 3.64 | 1.34 | 4.07 | 1.20 | -5.68 | <.001 | -.48 |
| Child_Safety_Composite | 133 | 5.05 | 1.09 | 5.08 | 0.95 | -0.36 | .722 | -.03 |
| Influ_Safety_Composite^a^ | 129 | 4.94 | 1.15 | 5.01 | 1.08 | -0.87 | .388 | -.08 |
| Child_Serious | 139 | 5.44 | 0.96 | 5.42 | 0.85 | 0.27 | .791 | .02 |
| Influ_Serious^b^ | 141 | 4.05 | 1.49 | 4.31 | 1.33 | -2.41 | .017 | -.20 |
| Trust_Composite | 136 | 5.05 | 1.21 | 5.29 | 1.03 | -3.72 | <.001 | -.32 |
| *Note*. Response scale: 1–6.  ^a^“The risk of side-effects outweighs the benefits of influenza vaccines” excluded  ^b^ The item has been reversed. | | | | | | | | |
