# Supplementary material for: Spillover effects of the COVID-19 pandemic on attitudes to influenza and childhood vaccines
Source: BMC Public Health. 2023 Apr 25;23:764. doi: 10.1186/s12889-023-15653-4 (PMC10126550; doi:10.1186/s12889-023-15653-4)
Supplement: Supplementary file 6 — Additional file 6. [file 12889_2023_15653_MOESM6_ESM.docx]

| **Table S6** |  |  |  |  |  |  |  |  |
| --- | --- | --- | --- | --- | --- | --- | --- | --- |
| *Means, Standard Deviations and Results of Paired-samples t-tests for Men and Women in Study 2* | | | | | | | | |
| Item |  |  |  |  |  |  |  |  |
|  | Men | | | | | | | |
|  |  | Pre-pandemic | | Mid-pandemic | |  |  |  |
|  | *n* | Mean | *SD* | Mean | *SD* | *t* | *p* | *d* |
| Child_Benefit_Composite | 43 | 3.79 | 0.25 | 3.76 | 0.35 | 0.82 | .413 | .13 |
| Influ_Hygiene^a^ | 44 | 2.96 | 0.75 | 2.93 | 0.93 | 0.18 | .855 | .03 |
| Influ_Effective | 44 | 3.00 | 0.61 | 3.18 | 0.79 | -1.48 | .146 | -.22 |
| Child_Safety_Composite^b^ | 41 | 3.70 | 0.31 | 3.81 | 0.33 | -2.11 | .041 | -.33 |
| Influ_Safety_Composite | 44 | 3.32 | 0.60 | 3.53 | 0.61 | -2.54 | .015 | -.38 |
| Child_Serious | 43 | 3.56 | 0.70 | 3.58 | 0.63 | -0.22 | .830 | -.03 |
| Influ_Serious^a^ | 44 | 3.11 | 0.92 | 3.46 | 0.82 | -2.10 | .042 | -.32 |
| Trust_Composite | 44 | 3.56 | 0.50 | 3.64 | 0.50 | -1.48 | .147 | -.22 |
|  | Women | | | | | | | |
|  |  | Pre-pandemic | | Mid-pandemic | |  |  |  |
|  | *n* | Mean | *SD* | Mean | *SD* | *t* | *p* | *d* |
| Child_Benefit_Composite | 138 | 3.71 | 0.35 | 3.72 | 0.35 | -35 | .729 | -.03 |
| Influ_Hygiene^a^ | 146 | 2.73 | 0.90 | 2.97 | 0.93 | -2.98 | .003 | -.25 |
| Influ_Effective | 146 | 2.67 | 0.79 | 2.99 | 0.76 | -4.42 | <.001 | -.37 |
| Child_Safety_Composite^b^ | 136 | 3.51 | 0.56 | 3.69 | 0.45 | -5.47 | <.001 | -.47 |
| Influ_Safety_Composite | 142 | 3.05 | 0.82 | 3.41 | 0.72 | -6.22 | <.001 | -.52 |
| Child_Serious | 146 | 3.60 | 0.67 | 3.66 | 0.61 | -0.98 | .331 | -.08 |
| Influ_Serious^a^ | 145 | 3.23 | 0.78 | 3.34 | 0.83 | -1.70 | .092 | -.14 |
| Trust_Composite | 145 | 3.44 | 0.64 | 3.46 | 0.65 | -0.57 | .573 | -.05 |
| *Note*. Response scale: 1–4.  ^a^ The item has been reversed.  ^b^ “The risk of side-effects outweighs the benefits of childhood vaccines” excluded. | | | | | | | | |
